# Supplementary material for: Integrating AI with Advanced Hyperspectral Imaging for Enhanced Classification of Selected Gastrointestinal Diseases
Source: Bioengineering (Basel). 2025 Aug 8;12(8):852. doi: 10.3390/bioengineering12080852 (PMC12383326; doi:10.3390/bioengineering12080852)
Supplement: Supplementary file 1 [file bioengineering-12-00852-s001.zip › bioengineering-3770456-supplementary.pdf]

1. Gastrointestinal (GI) disease introduction

Early and accurate detection of gastrointestinal (GI) diseases—such as ulcerative colitis, polyps, and esophagitis—is vital for improving patient outcomes and reducing global mortality. Traditional white light imaging (WLI) used in endoscopy often lacks sufficient contrast to distinguish subtle mucosal and vascular abnormalities. To address this limitation, we propose an enhanced imaging approach using the Spectrum-Aided Vision Enhancer (SAVE), which converts WLI into hyperspectral imaging (HSI) representations that mimic narrow-band imaging (NBI) with superior tissue differentiation. SAVE images were used to train deep learning models for disease classification across four categories: normal, ulcerative colitis, polyps, and esophagitis. We evaluated three models— InceptionV3, YOLOv8x, and VGG16—on both WLI and SAVE images. Notably, InceptionV3 achieved an overall accuracy of 94% on both imaging types, but SAVE showed improved class-specific metrics, including 99% recall for ulcerative colitis and 99% precision for polyps. VGG16 demonstrated a 6% accuracy gain using SAVE (91% vs. 85% WLI), and YOLOv8x yielded a 10% gain (89% vs. 79%). Structural Similarity Index (SSIM) analysis confirmed a 94.27% match between SAVE and Olympus NBI images. These findings validate SAVE as a practical, cost-effective enhancement for standard WLI, enabling AI-assisted GI diagnostics with greater accuracy and clinical reliability.

2. YOLOv8x Model Training Results

2.1 YOLOv8x of WLI Images

2.1.1 Training and validation dataset of WLI

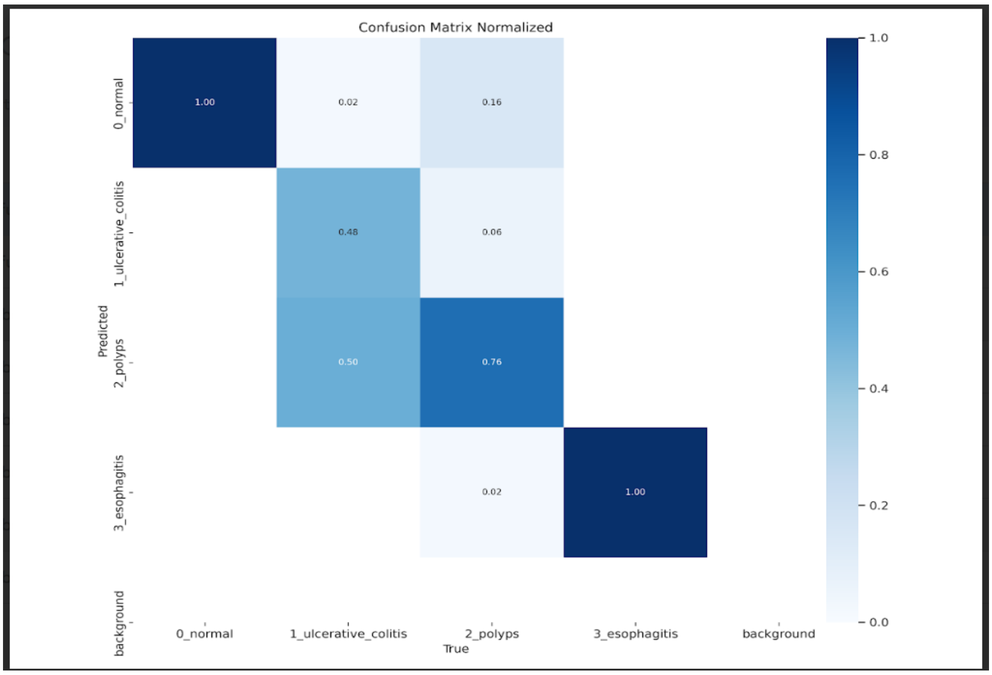

Figure S1. Confusion matrix representing the proportion of correct and incorrect predictions for each class of WLI Images.

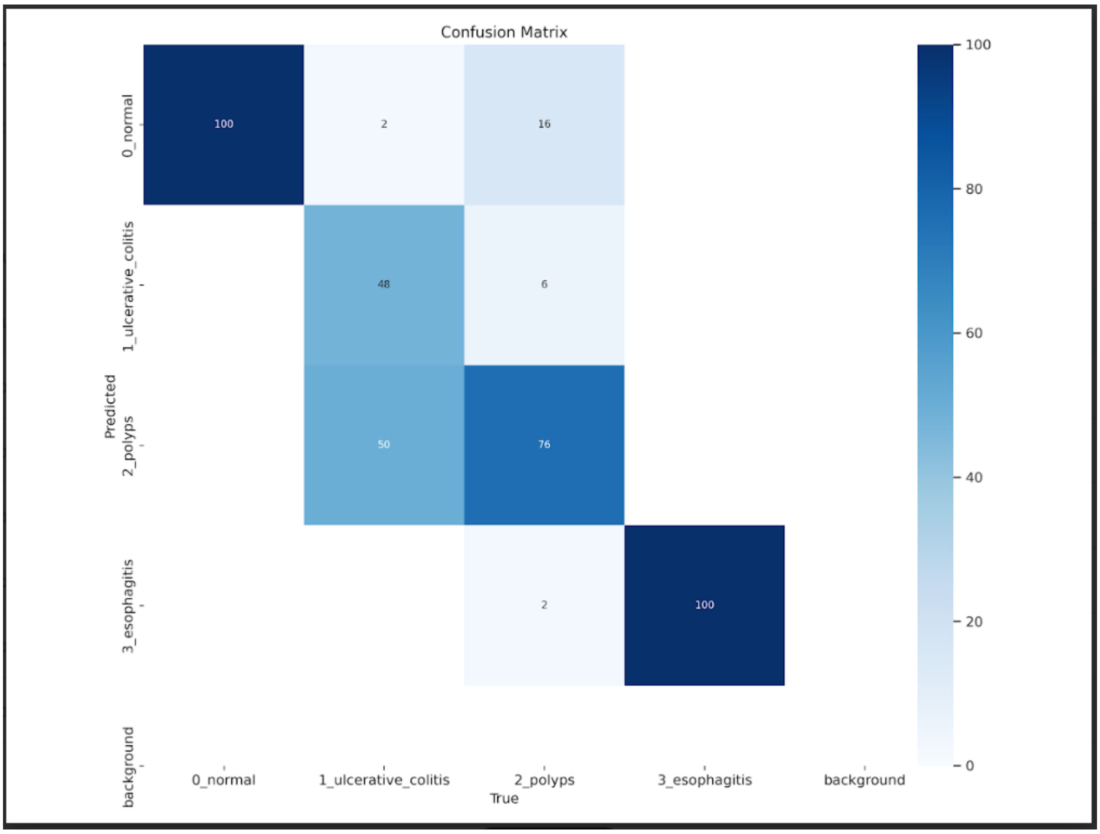

Figure S2. Confusion matrix showing the absolute number of predictions for each class of WLI Images.

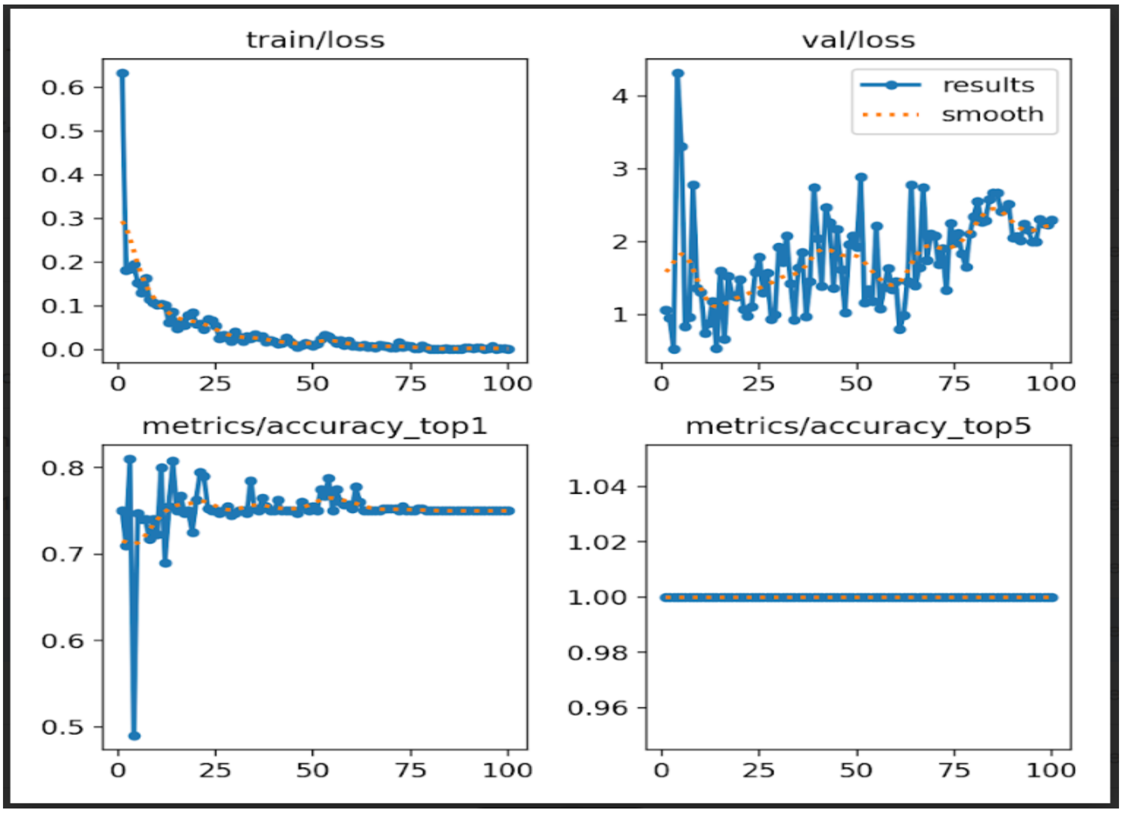

Figure S3. The training accuracy and loss of the YOLOv8x model for WLI images.

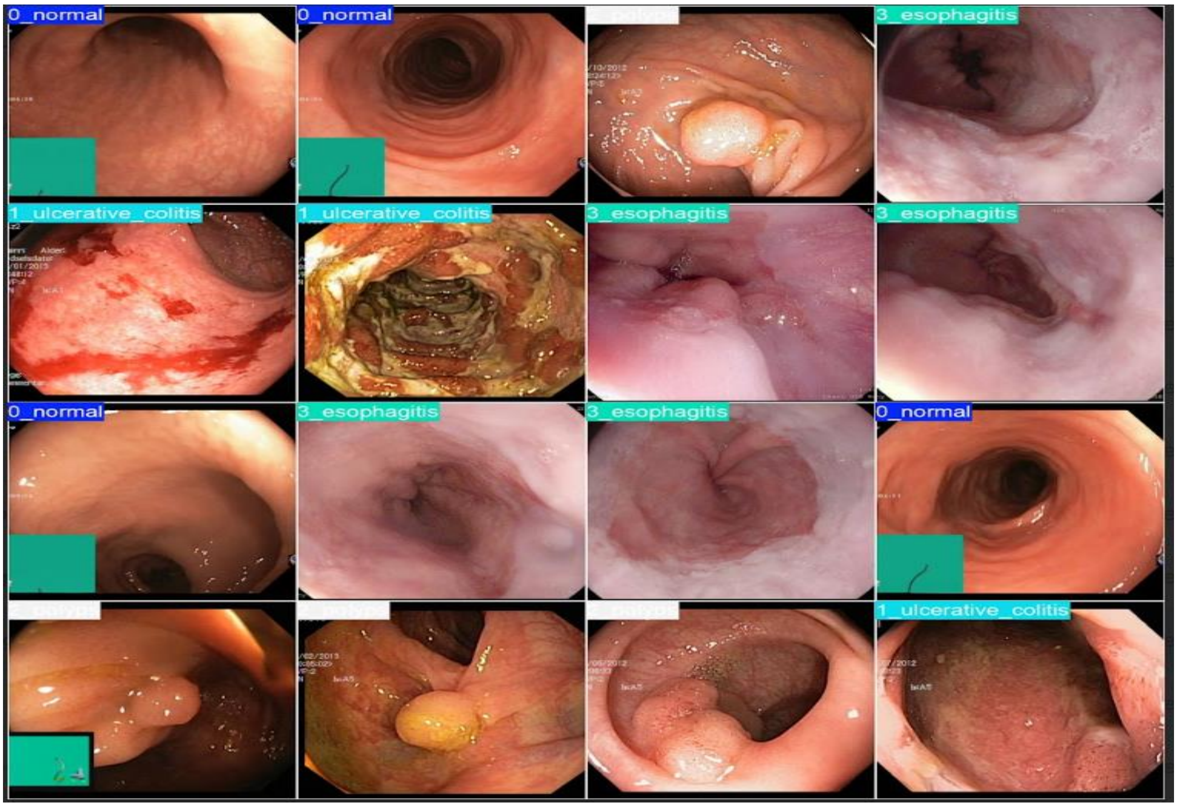

Figure S4. The labeled classes of WLI images over the validation dataset.

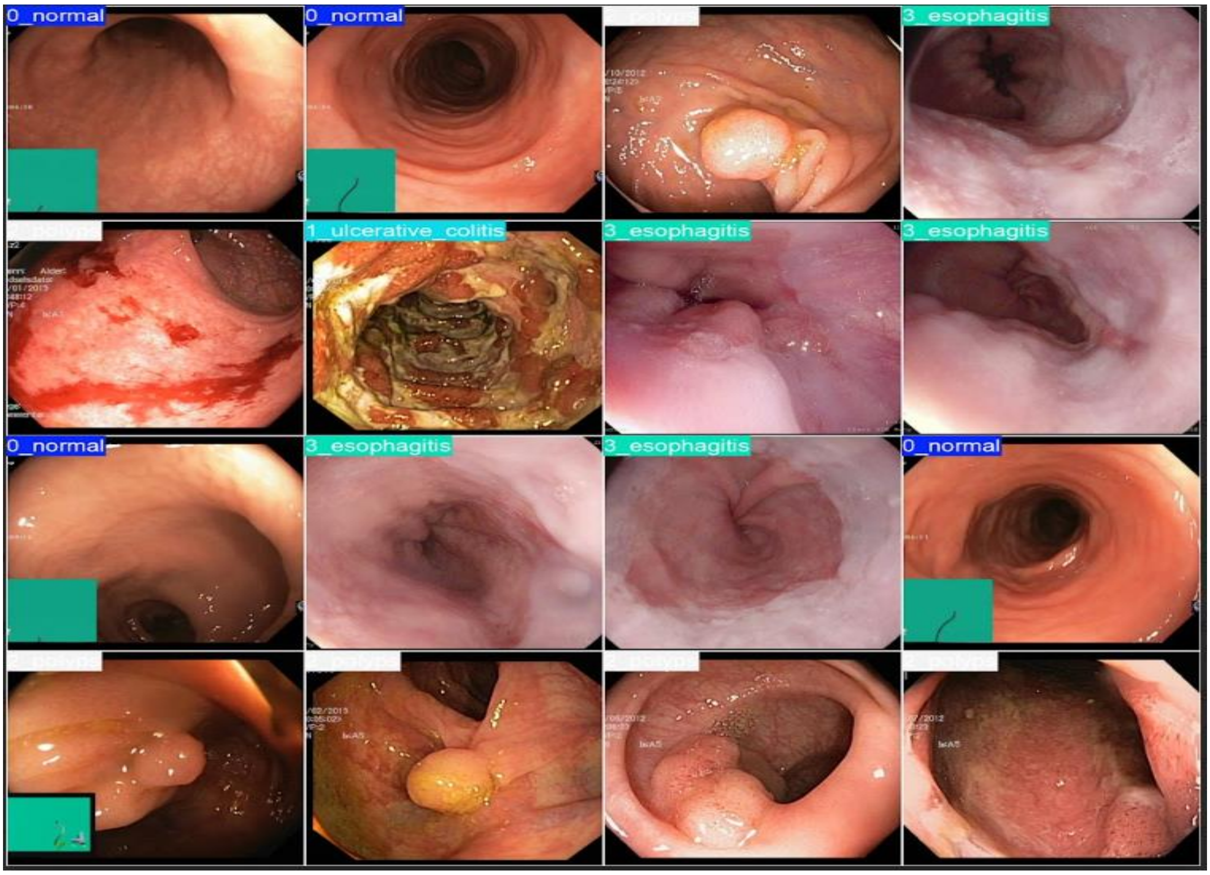

Figure S5. The predicted classes of WLI images over the validation dataset.

2.1.2 Test dataset of WLI

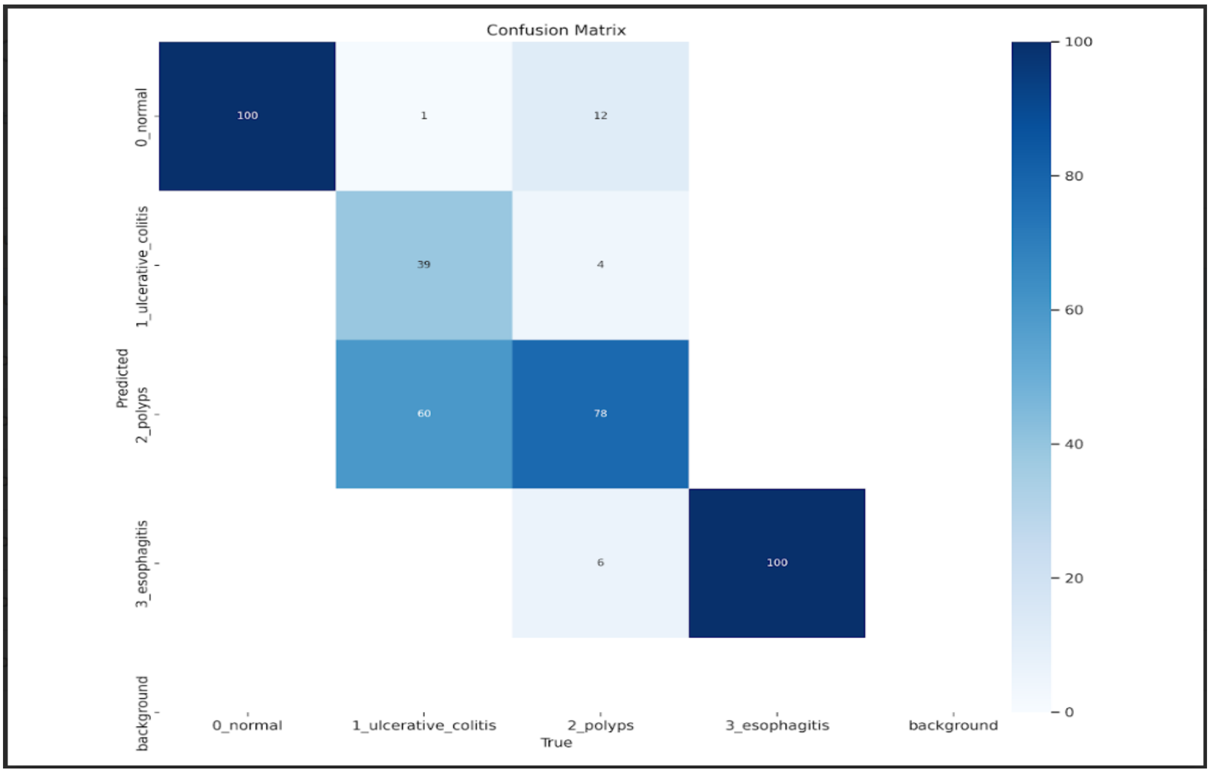

Figure S6. Confusion matrix showing the absolute number of predictions for each class of WLI Images over the Test dataset.

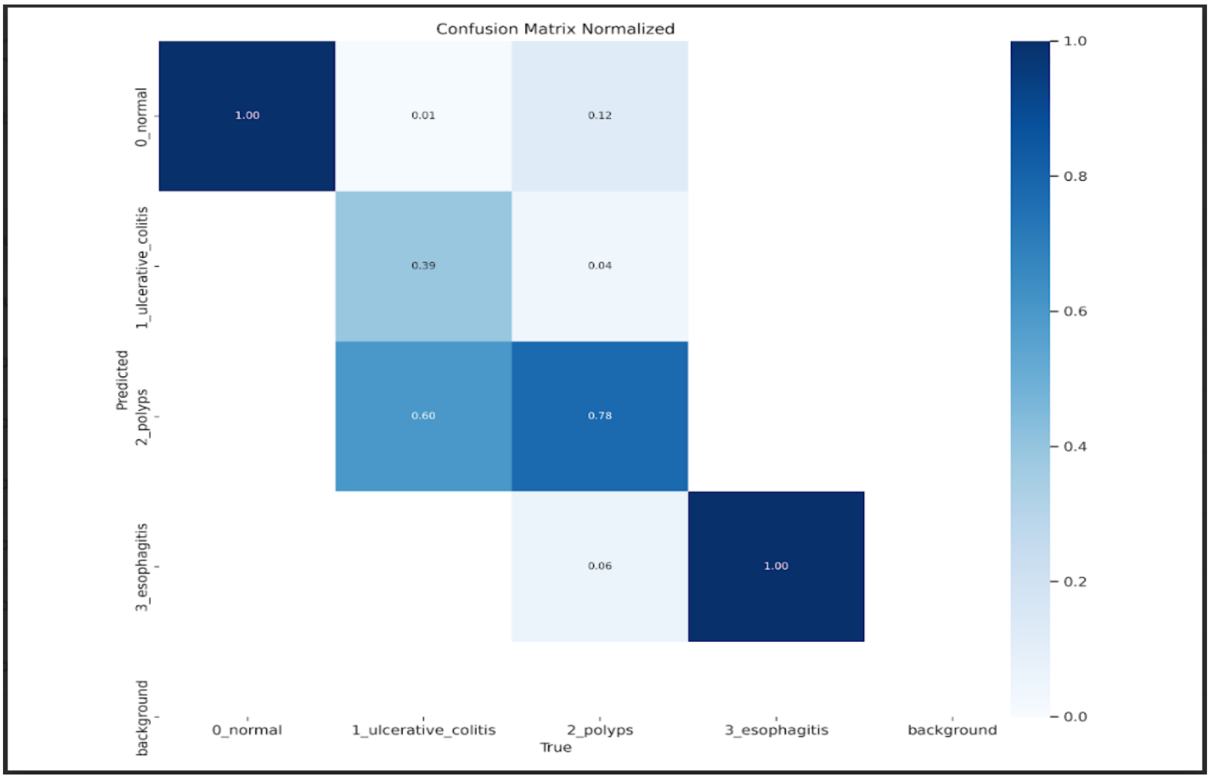

Figure S7. Confusion matrix representing the proportion of correct and incorrect predictions for each class of WLI Images over the Test dataset.

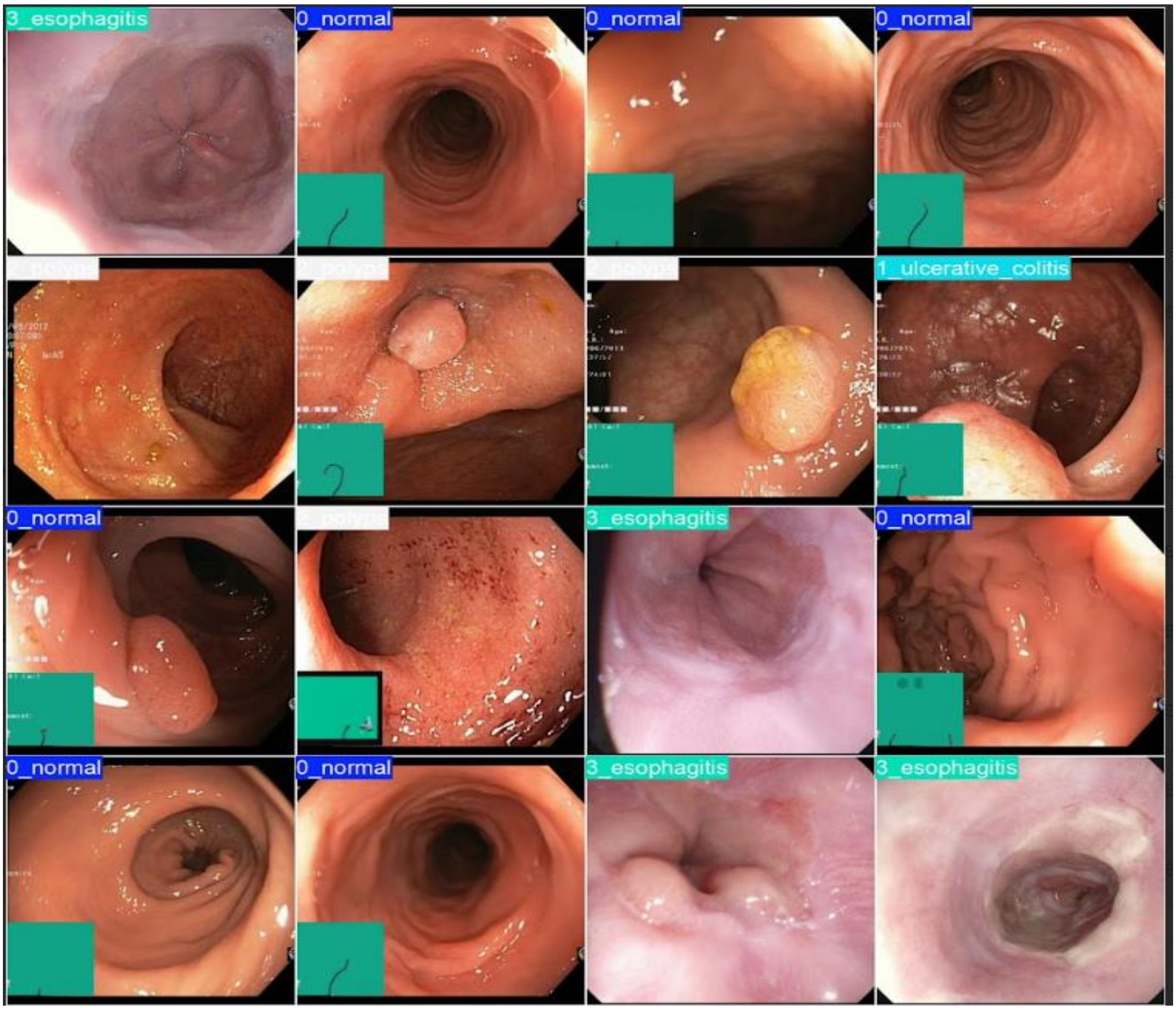

Figure S8. The predicted classes of the trained model over the test dataset for WLI Images.

2.2 YOLOv8X of SAVE Images

2.2.1 Training and validation dataset of SAVE

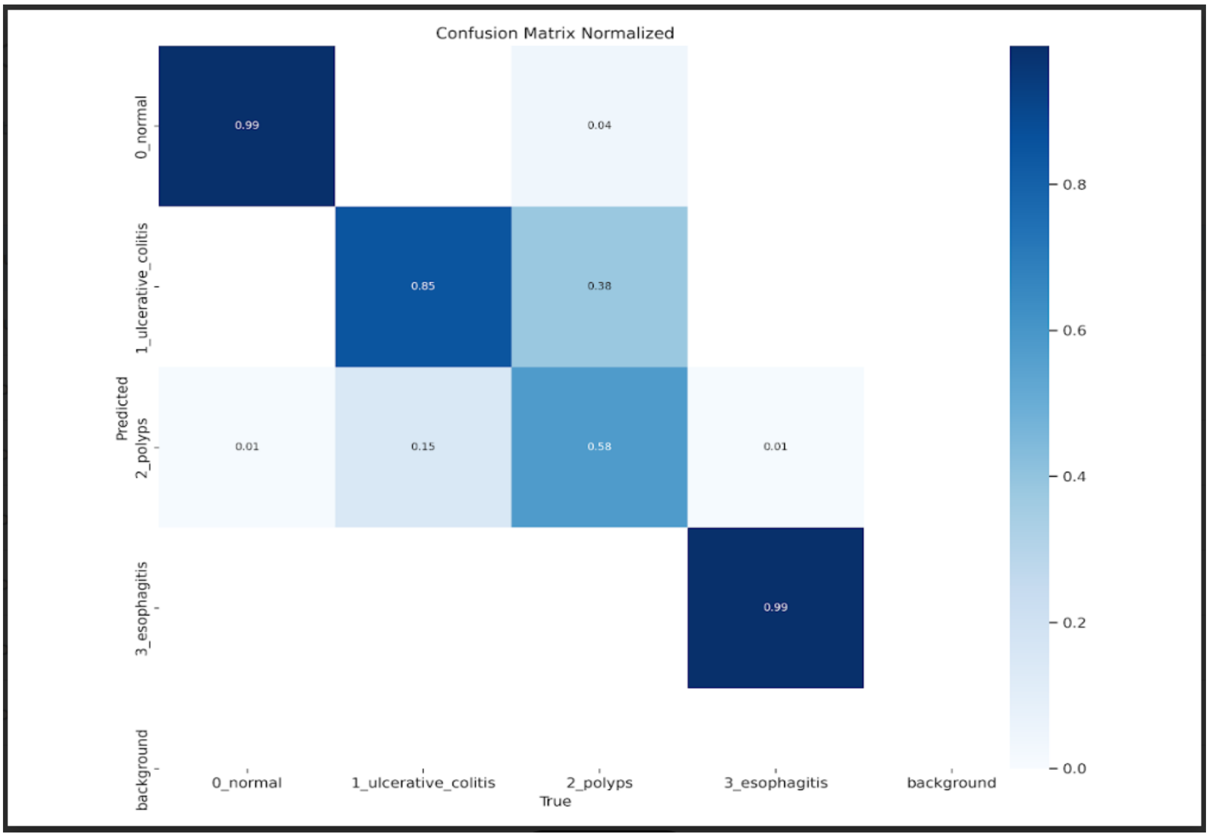

Figure S9. Confusion matrix representing the proportion of correct and incorrect predictions for each class of SAVE Images.

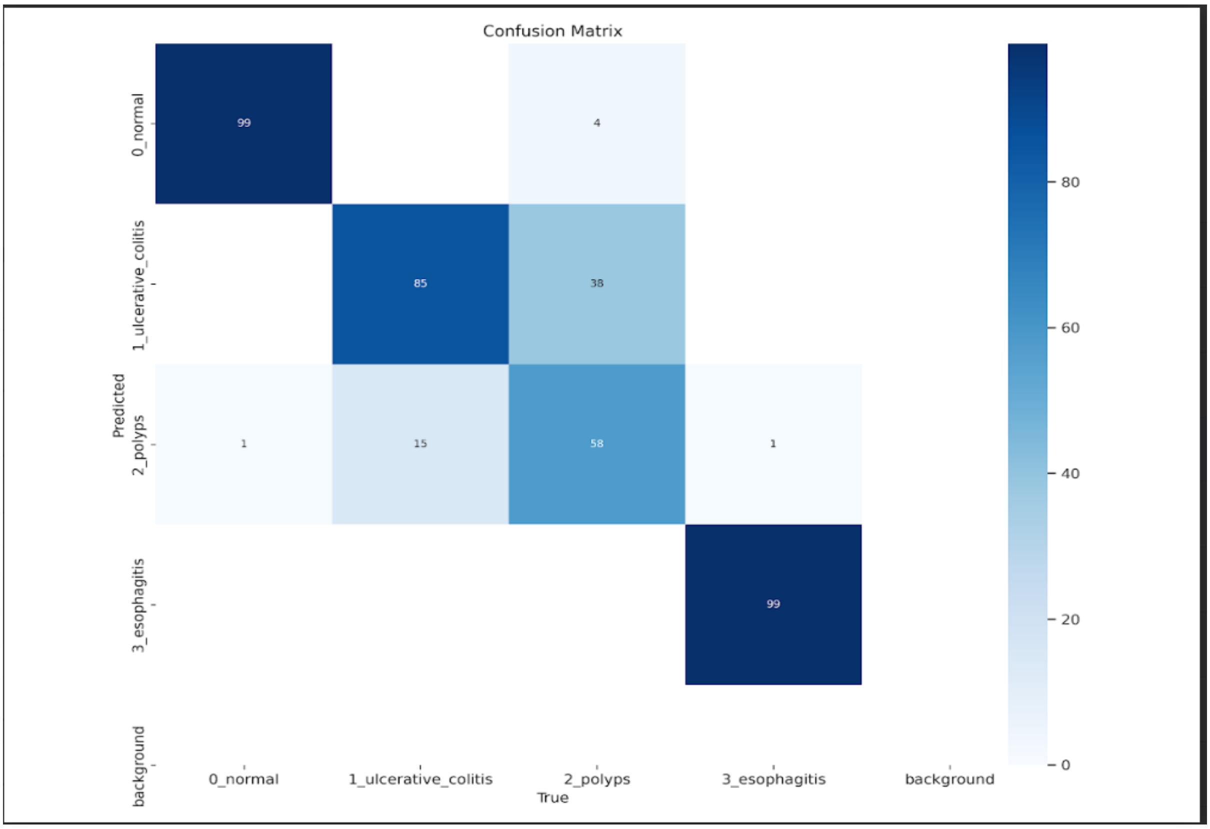

Figure S10. Confusion matrix showing the absolute number of predictions for each class of SAVE Images.

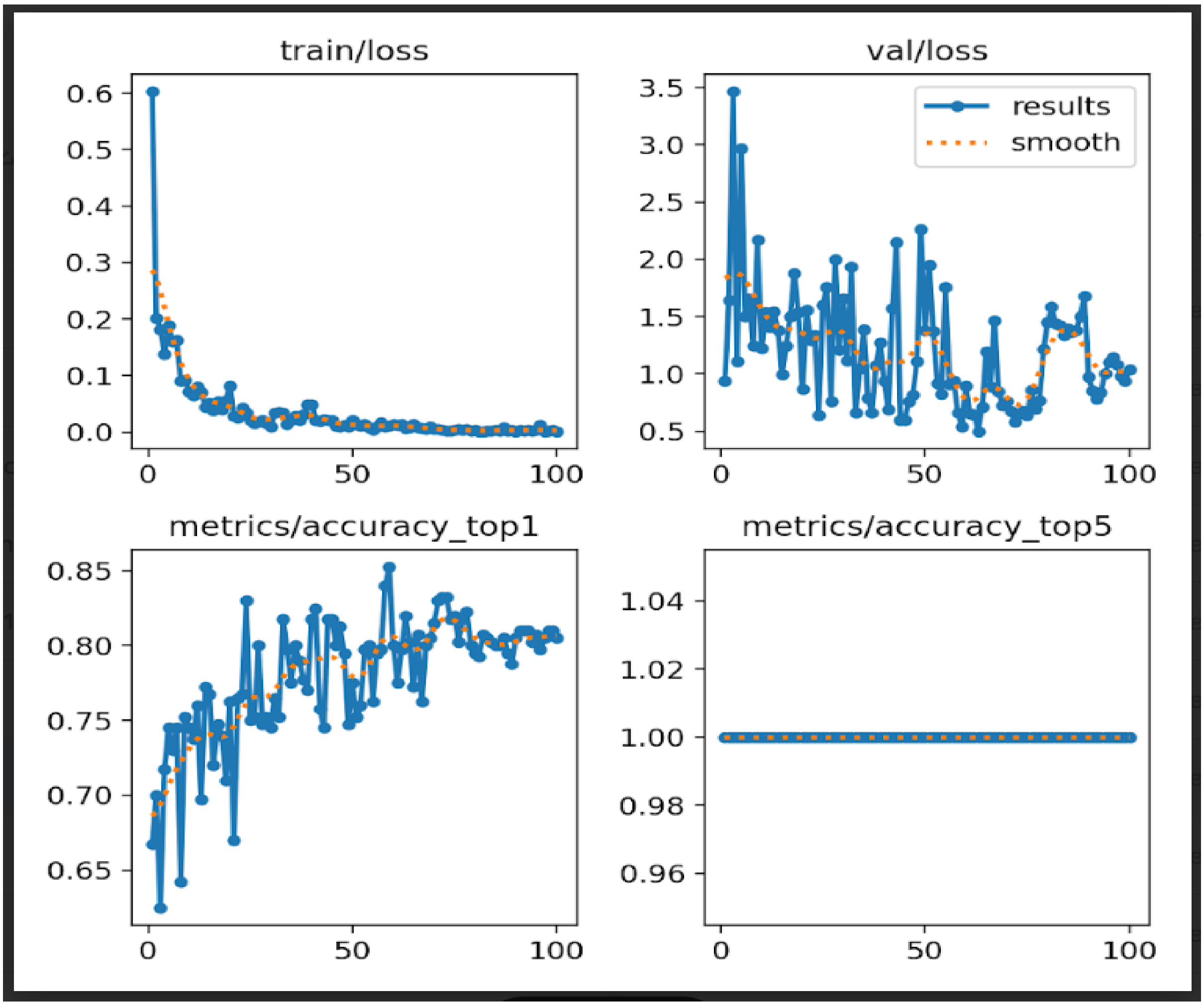

Figure S11. The accuracy and loss value of the training model for SAVE Images.

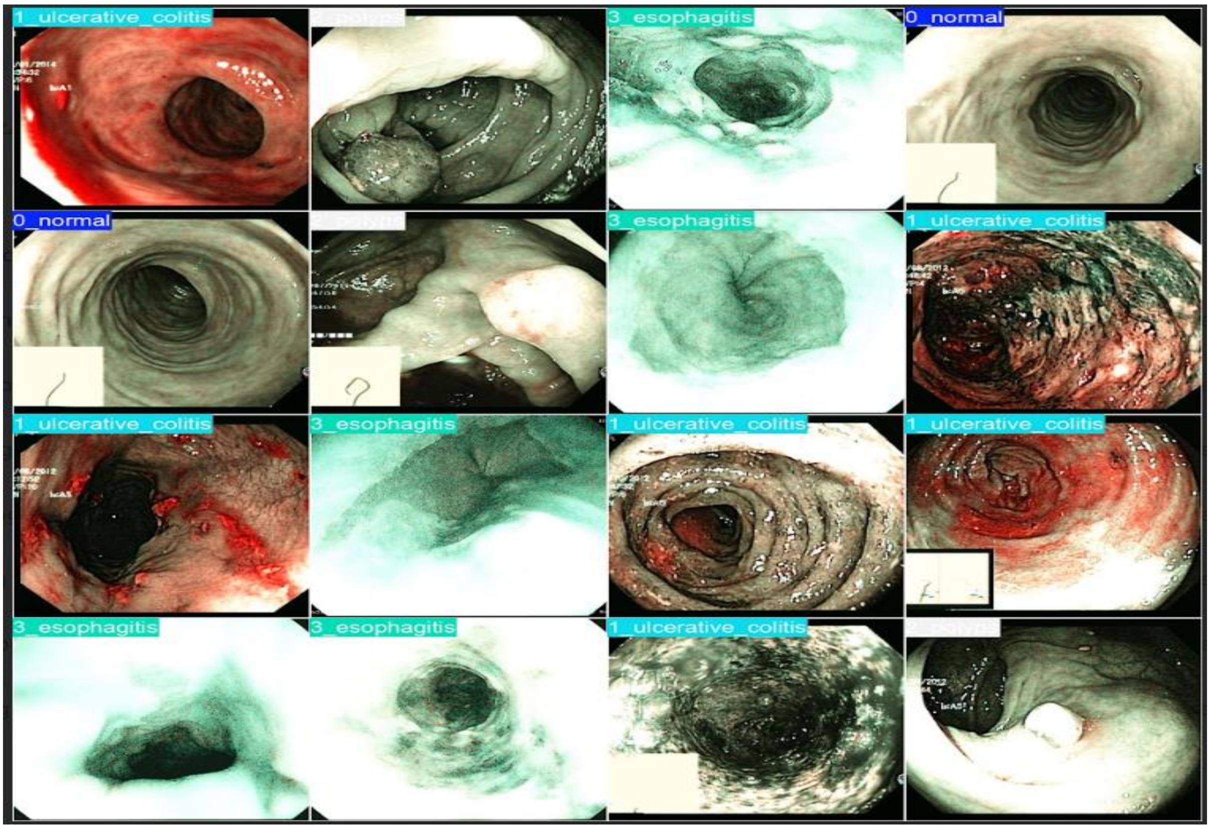

Figure S12. The labeled classes of SAVE images for the model training purpose.

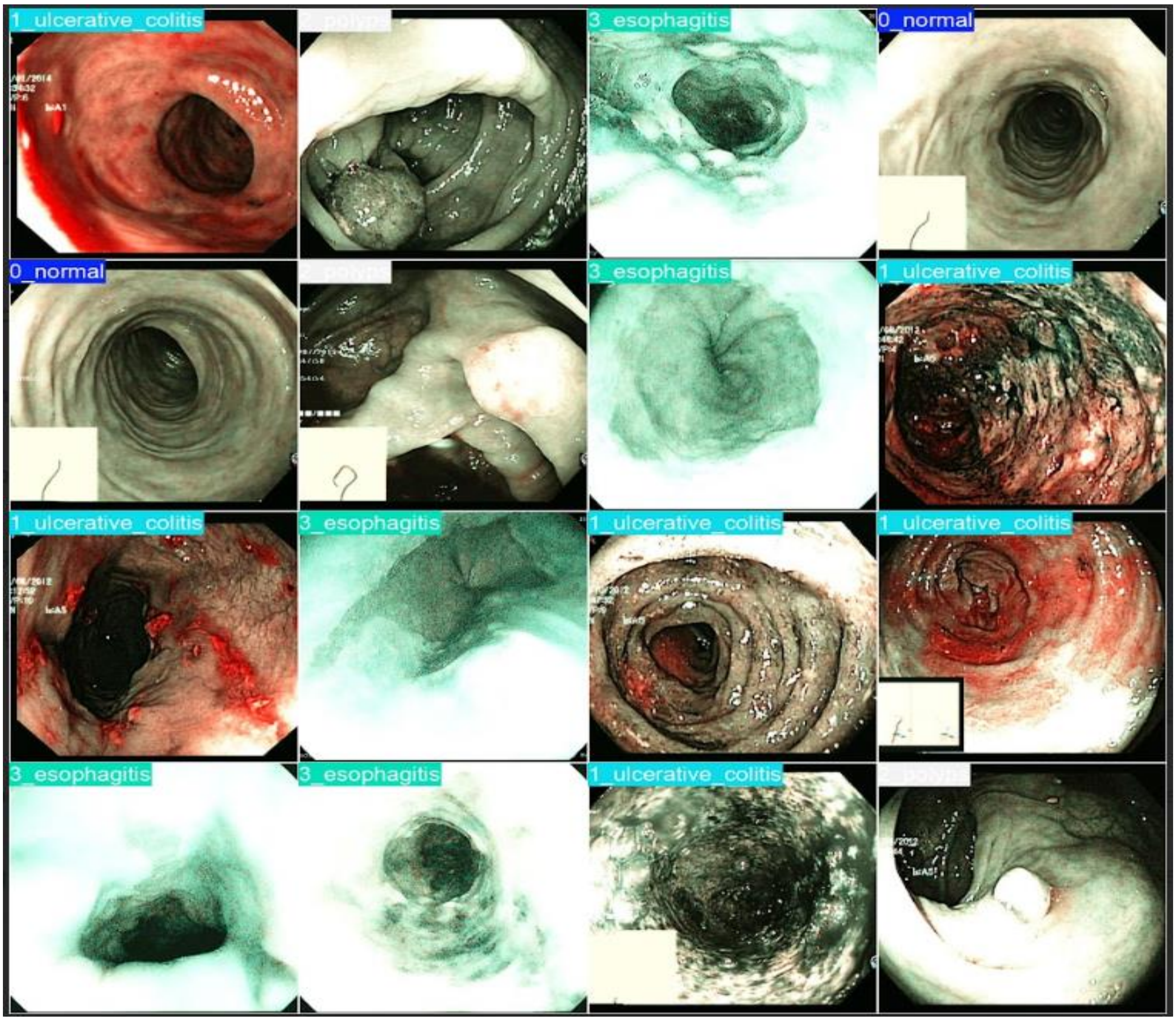

Figure S13. The predicted classes of SAVE images after the model has been trained.

2.2.2 Test dataset of SAVE

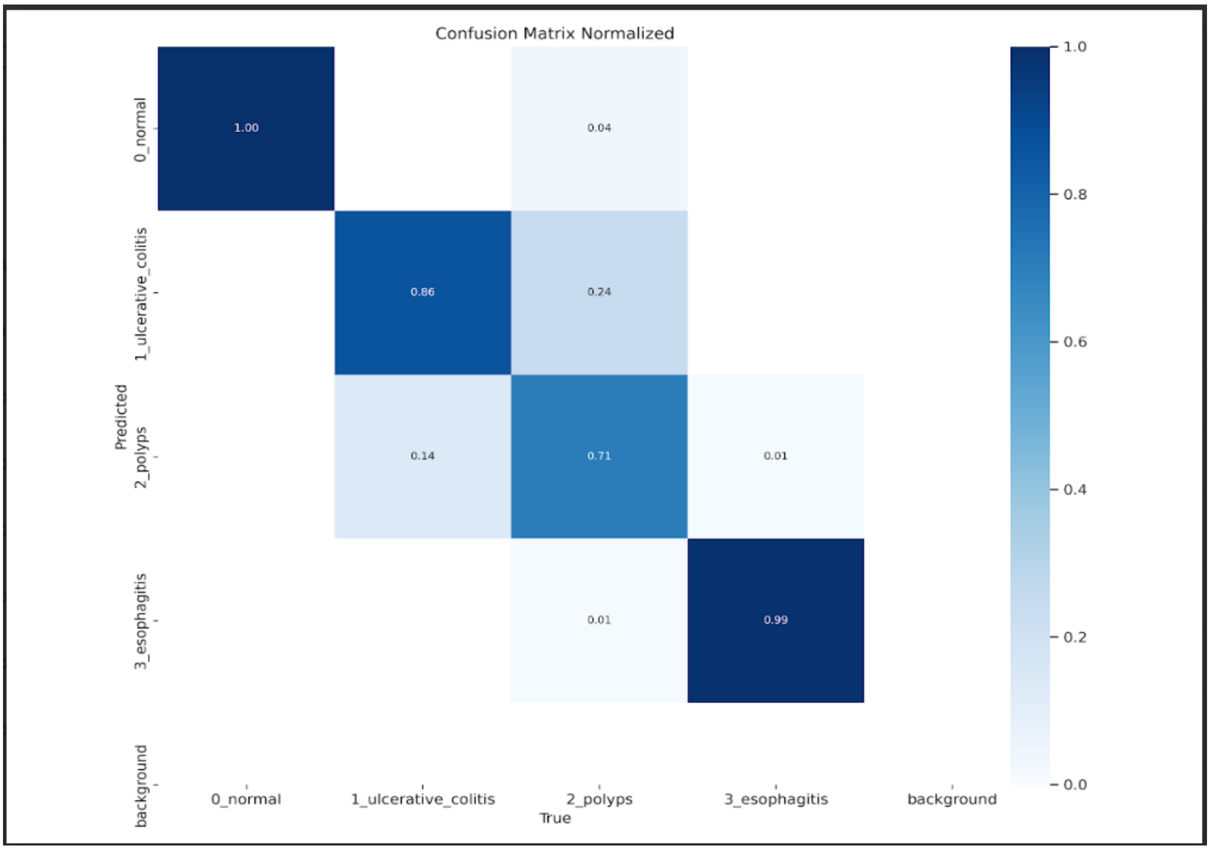

Figure S14. Confusion matrix representing the proportion of correct and incorrect predictions for each class of SAVE images over the Test dataset

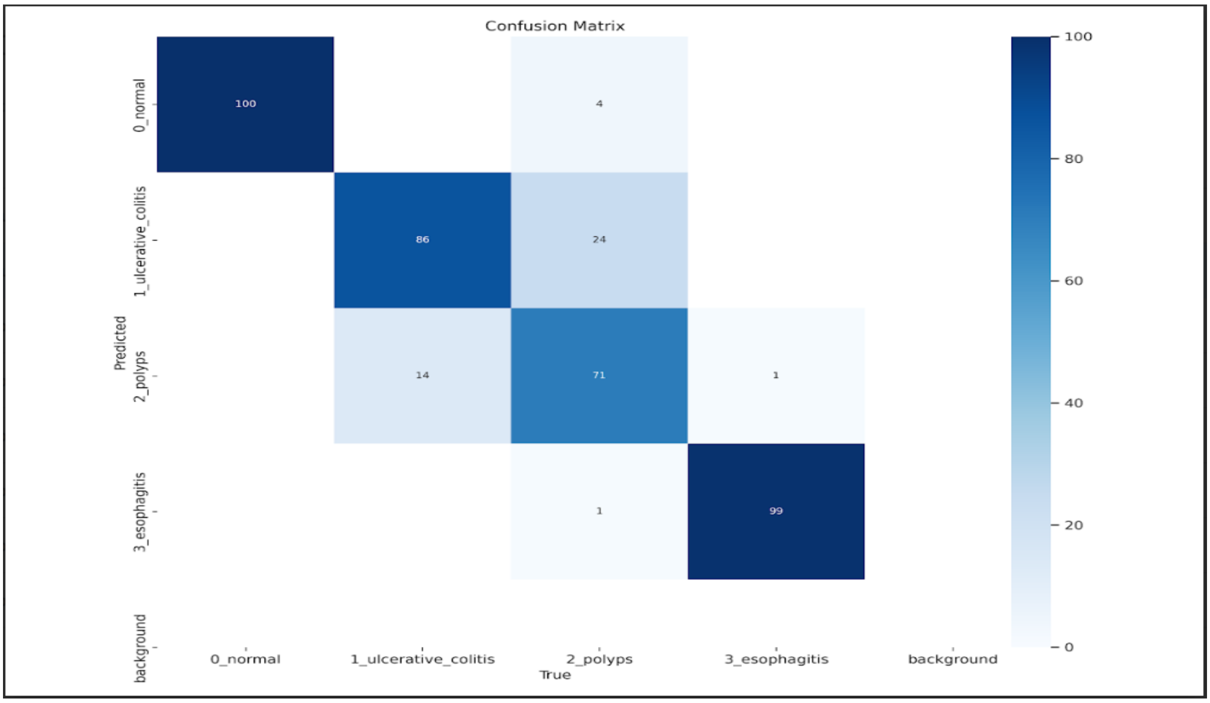

Figure S15. Confusion matrix showing the absolute number of predictions for each class of SAVE Images over the Test dataset.

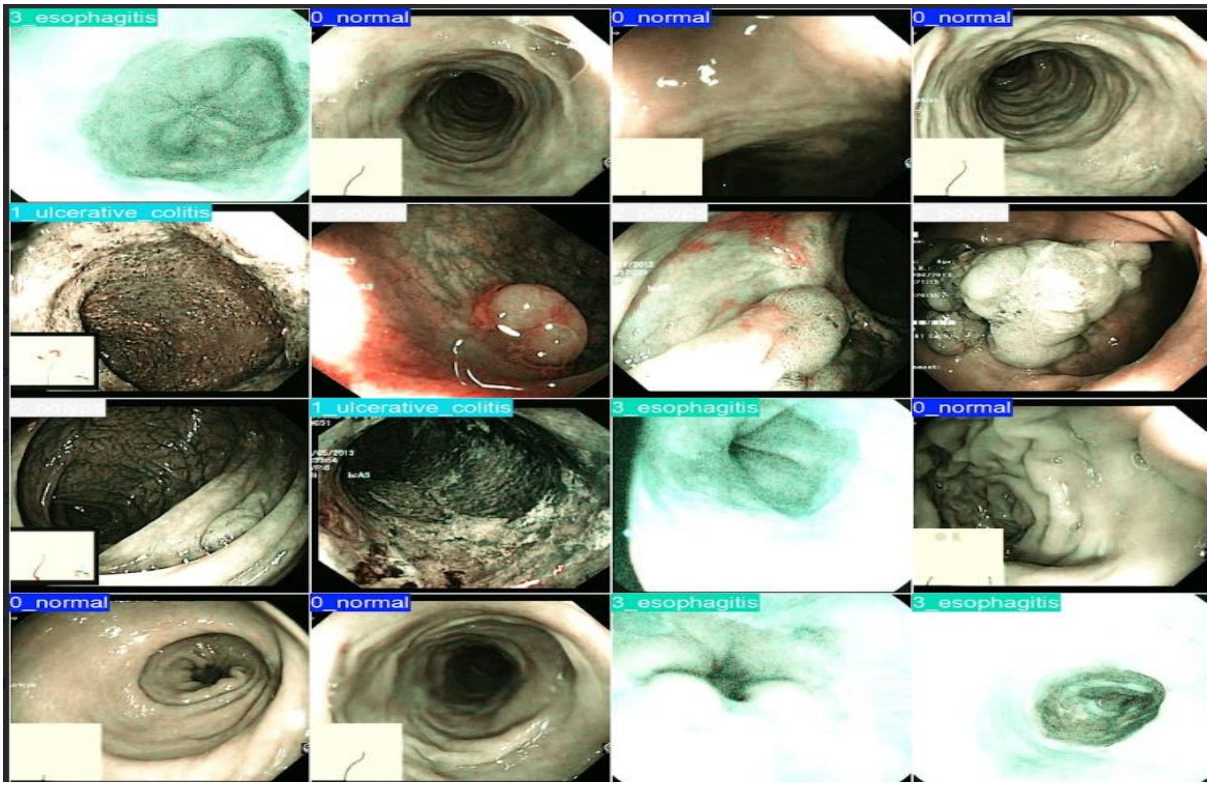

Figure S16. The labeled classes of SAVE images for training purposes.

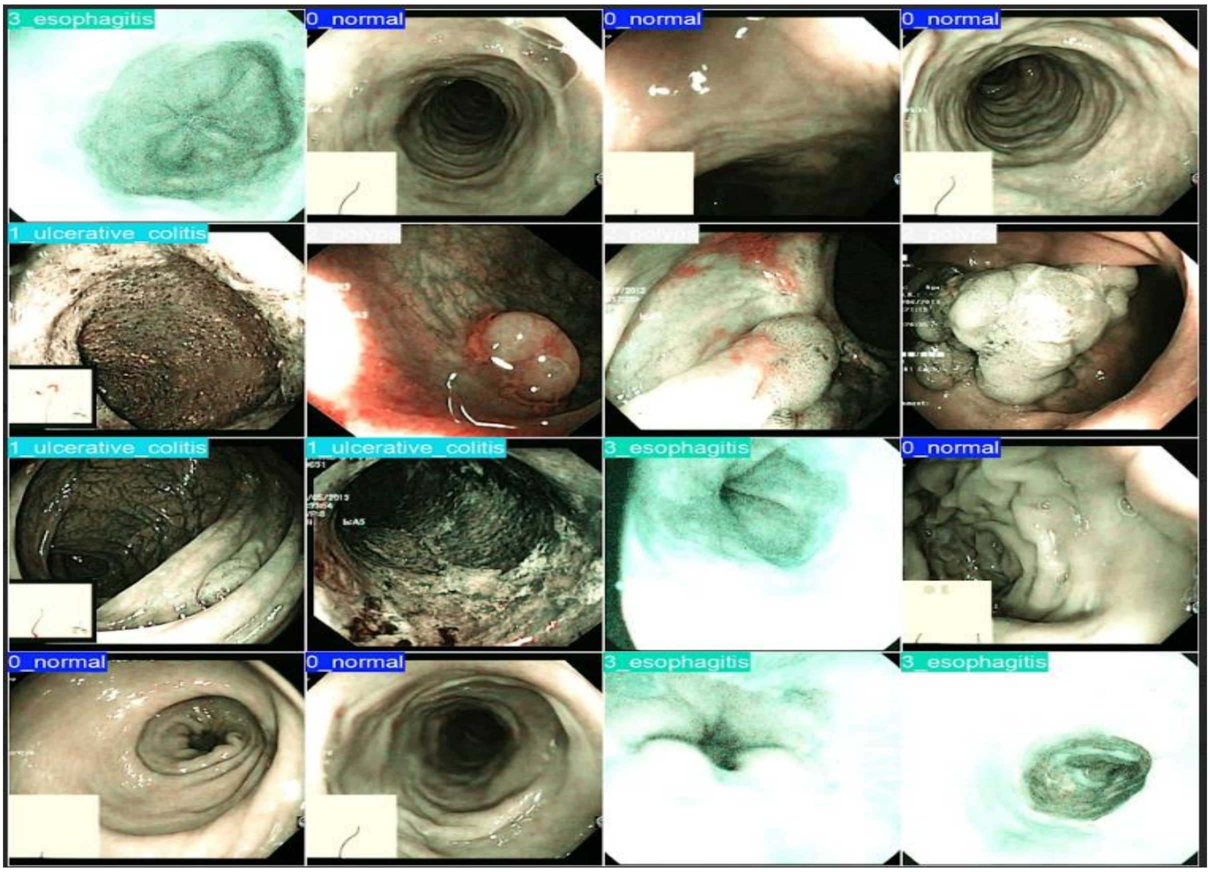

Figure S17. The predicted classes of the model over the test data of SAVE images.

3. Inceptinv3

3.1 WLI Images

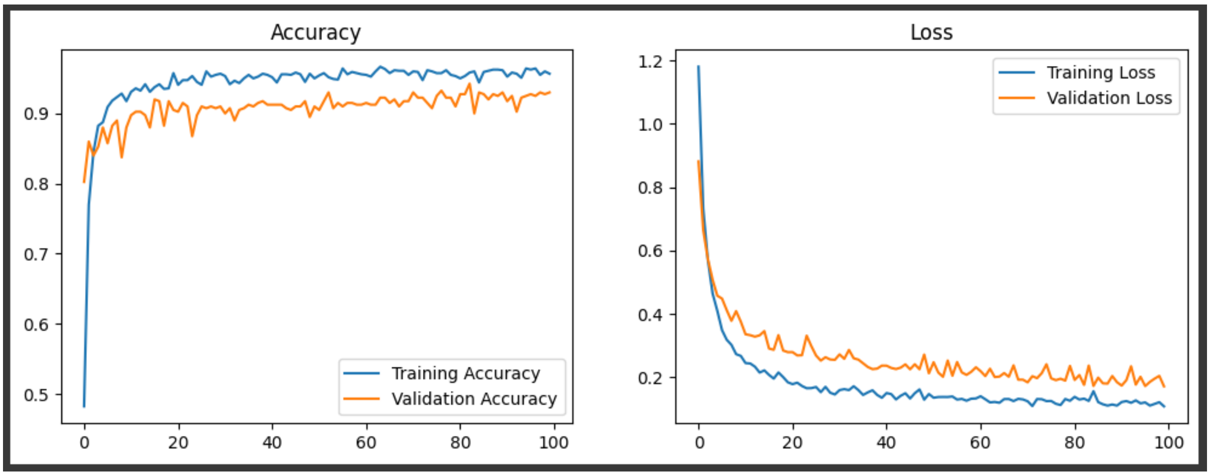

Figure S18. The accuracy and Loss value of the training and validation dataset.

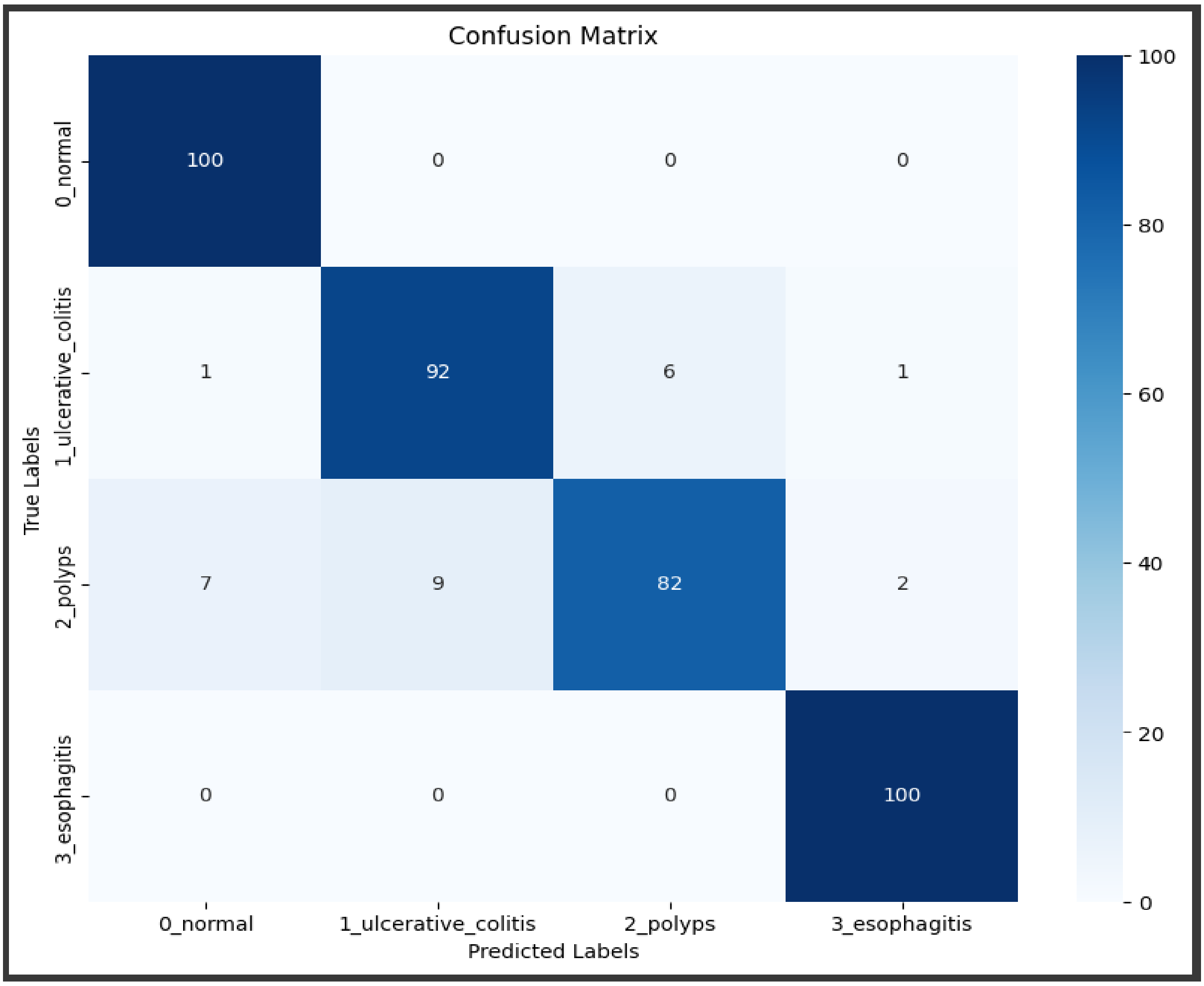

Figure S19. Confusion matrix showing the absolute number of predictions for each class of WLI Images.

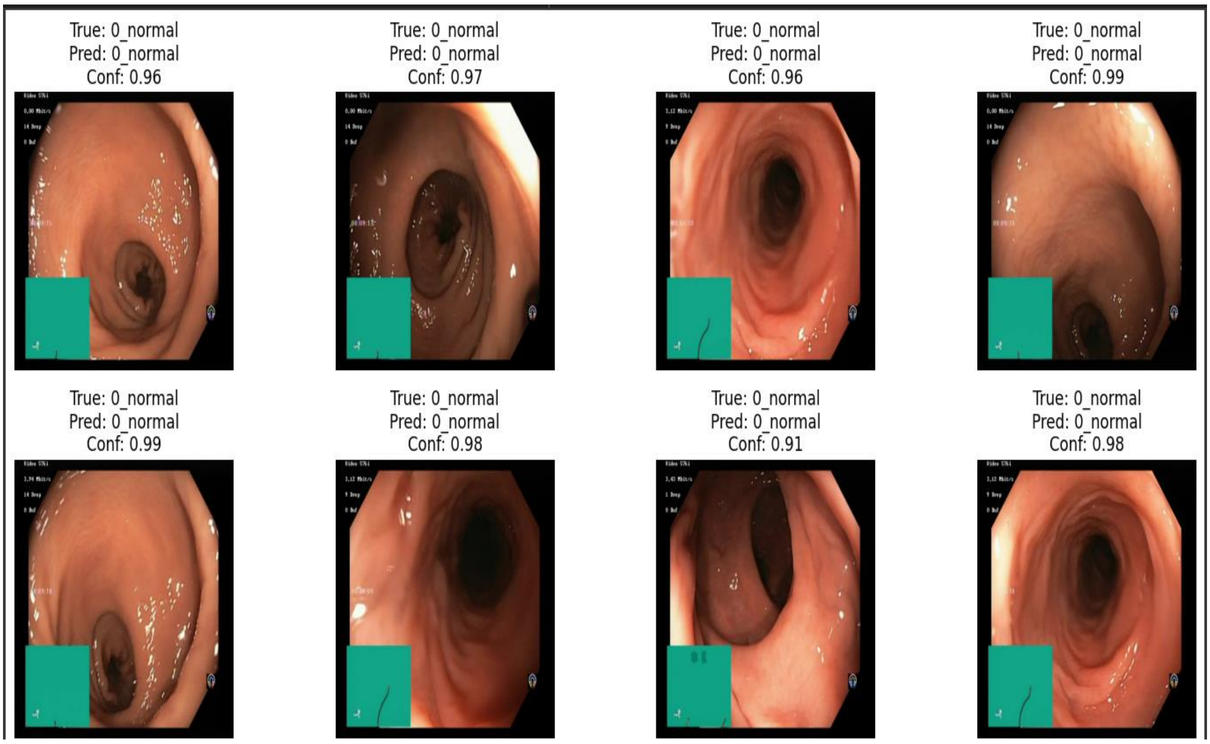

Figure S20. The prediction results of the training model for WLI Images.

3.2 SAVE IMAGES

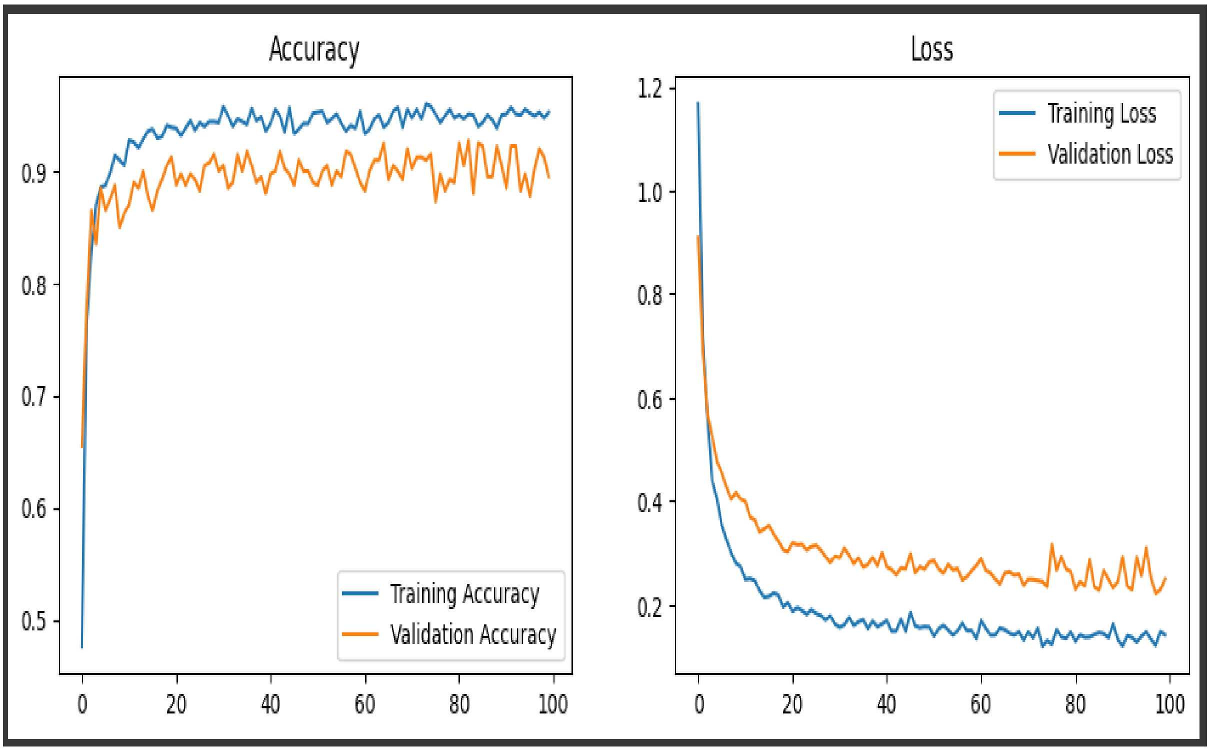

Figure S21. The accuracy and Loss value of the training and validation dataset of the SAVE dataset.

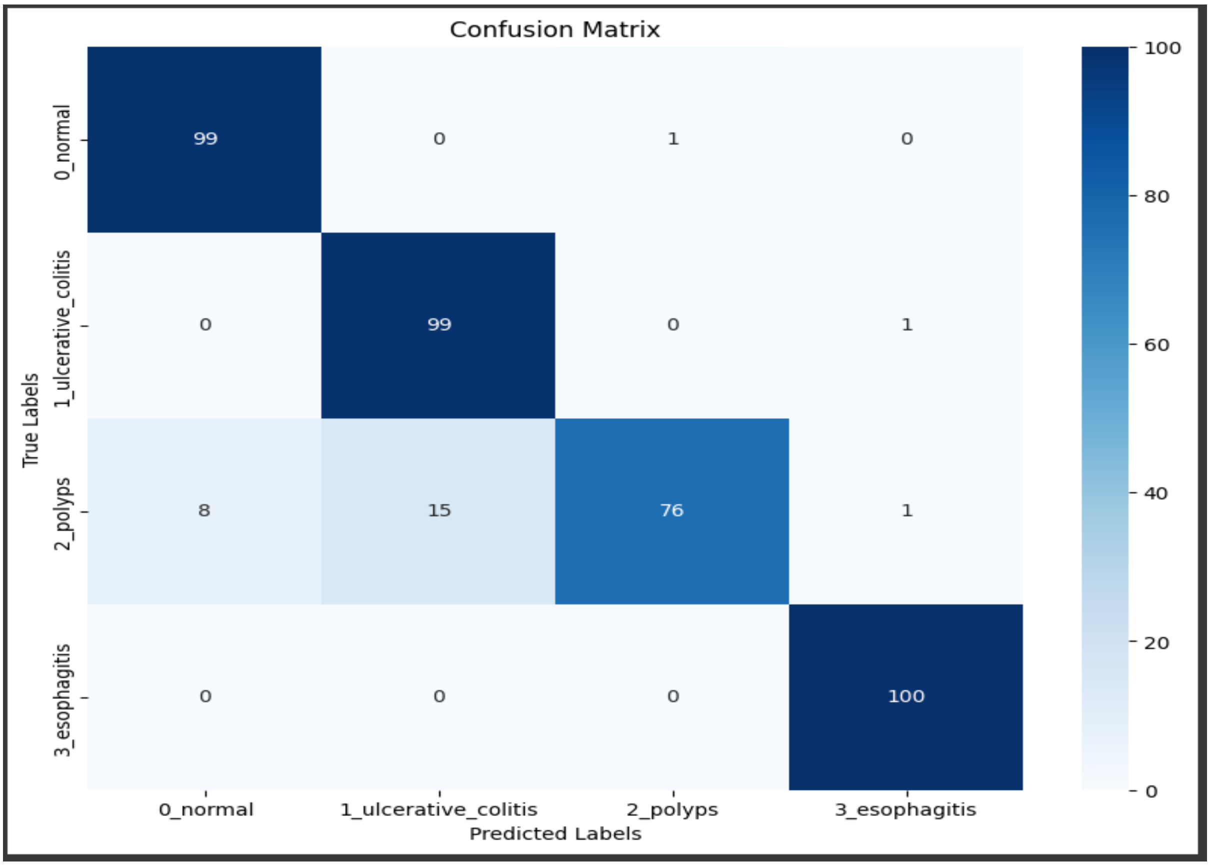

Figure S22. Confusion matrix showing the absolute number of predictions for each class of SAVE Images.

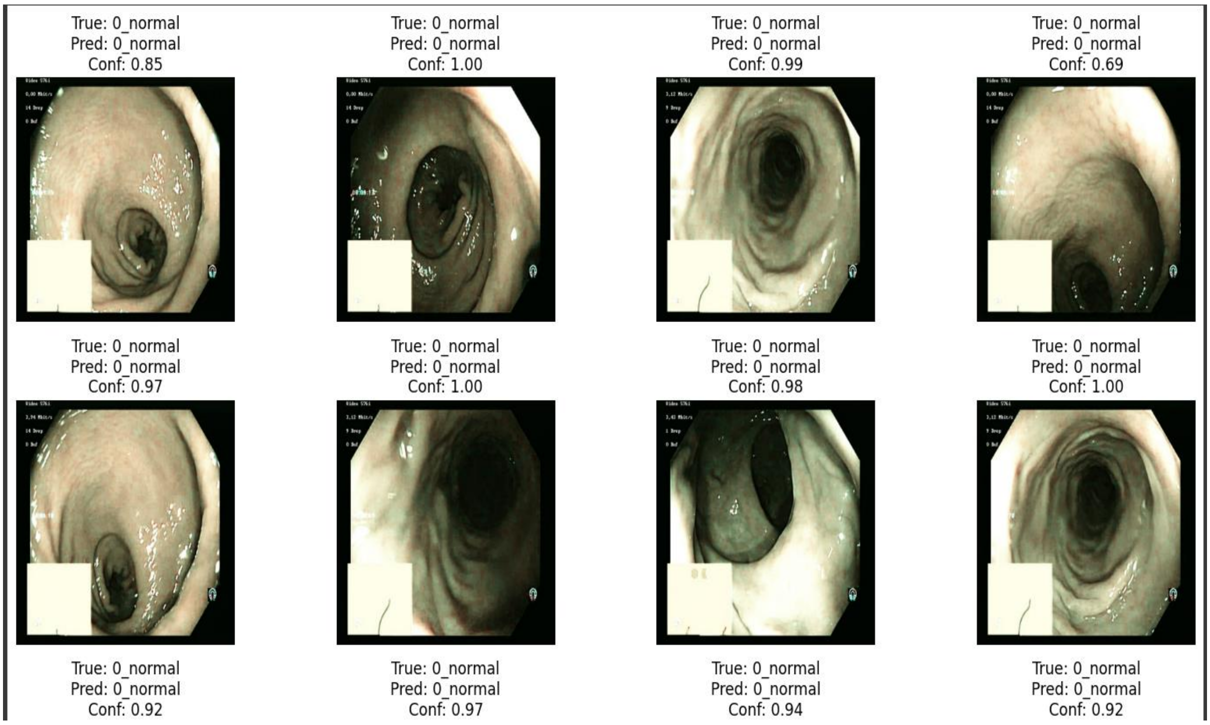

Figure S23. The prediction results of the training model for SAVE Images.

4. Vgg16

4.1 WLI Images

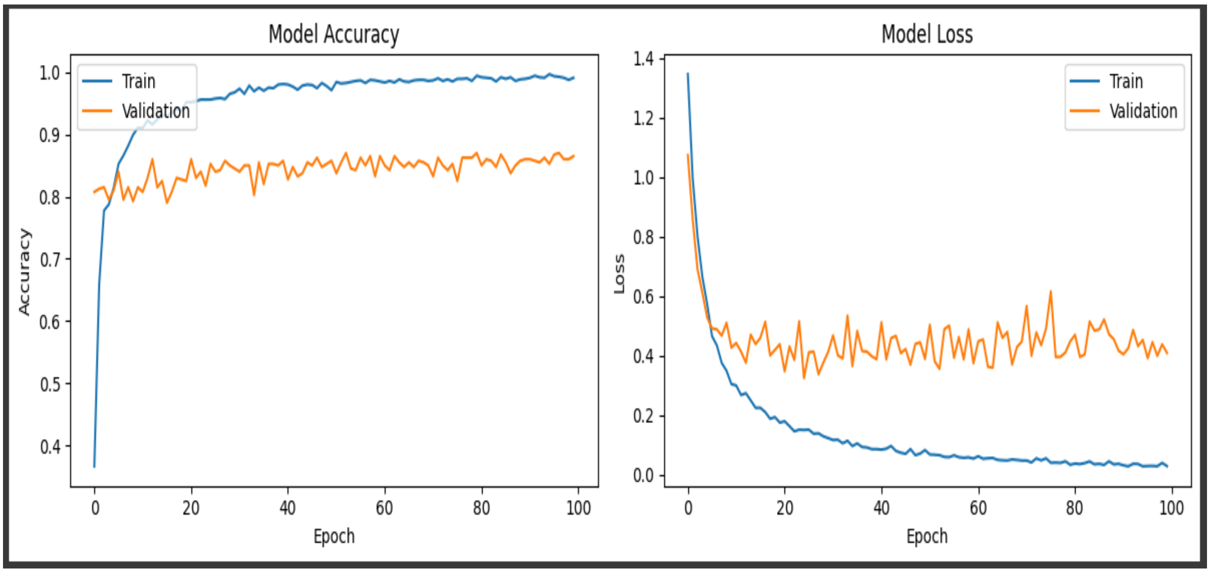

Figure S24 The accuracy and Loss value of the training and validation dataset of WLI dataset.

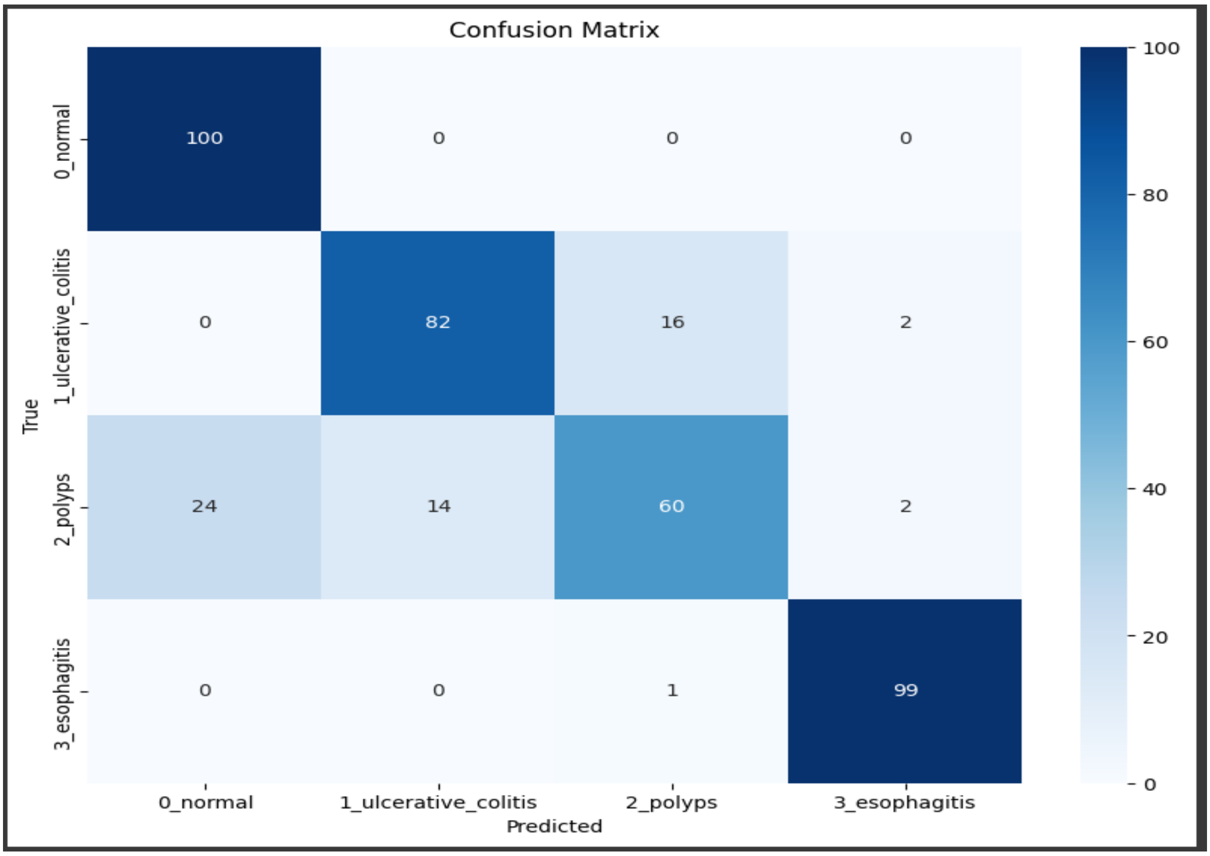

Figure S25 Confusion matrix showing the absolute number of predictions for each class of WLI Images.

4.2 SAVE Images

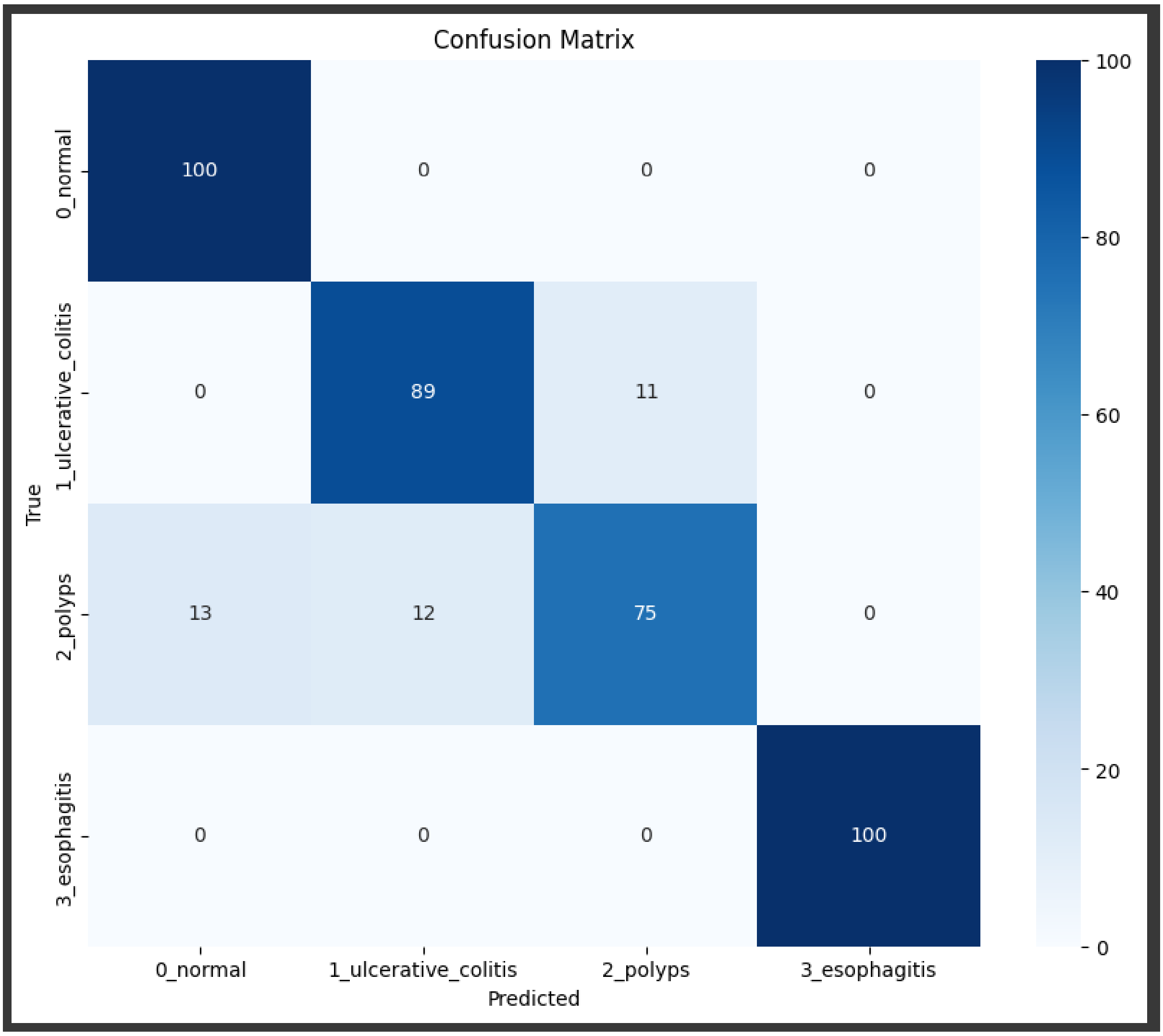

Figure S26 Confusion matrix showing the absolute number of predictions for each class of SAVE Images.

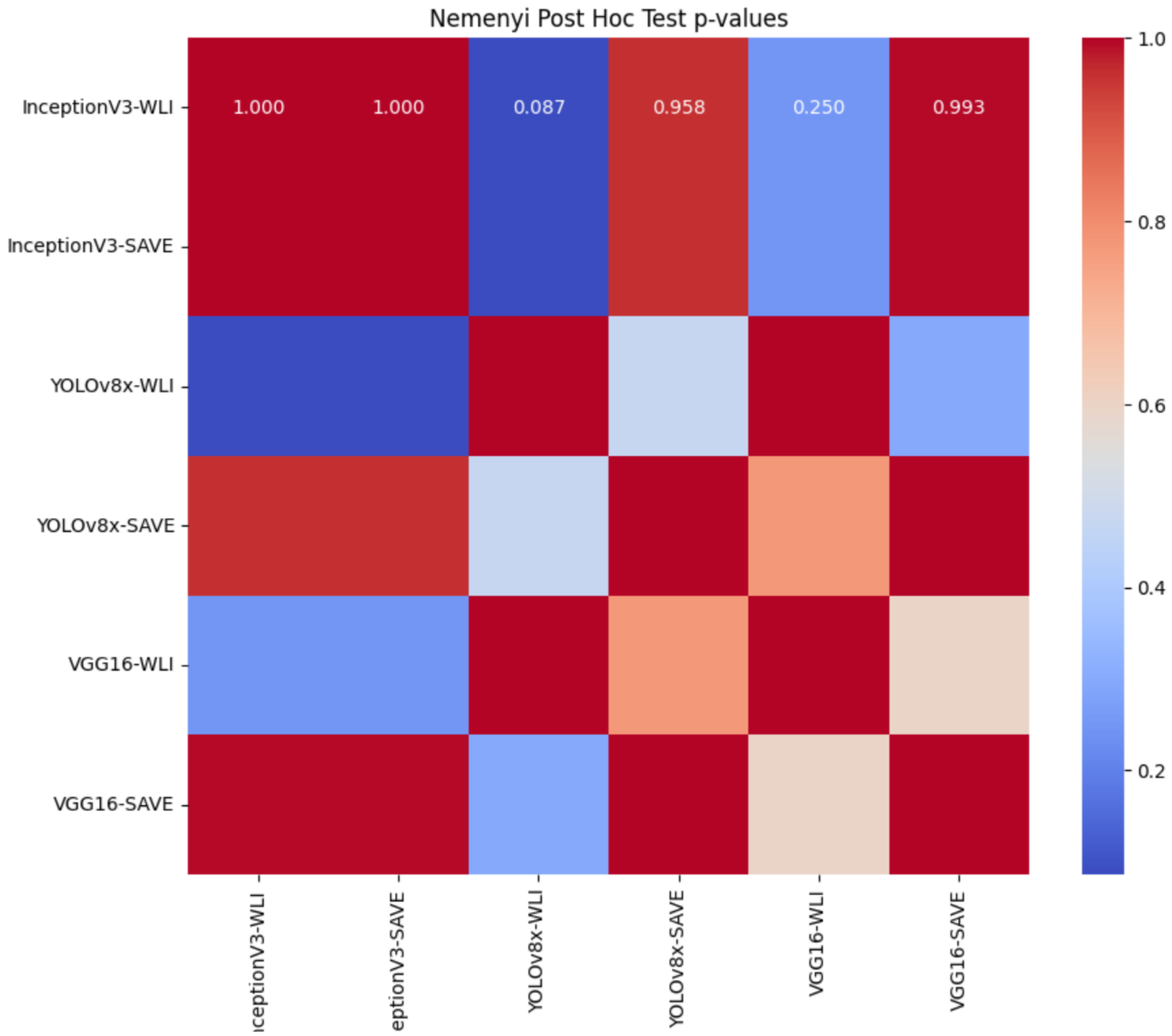

Figure S27. A heatmap visualizing the Nemenyi p-values has been included in the revised manuscript
